# Trans-Cellular Introduction of HIV-1 Protein Nef Induces Pathogenic Response in *Caenorhabditis elegans*

**DOI:** 10.1371/journal.pone.0015312

**Published:** 2010-12-13

**Authors:** Aamir Nazir, Shreesh Raj Sammi, Pankaj Singh, Raj Kamal Tripathi

**Affiliations:** 1 Laboratory of Functional Genomics and Molecular Toxicology, Division of Toxicology, Central Drug Research Institute (CSIR), Lucknow, India; 2 Laboratory of Immunotoxicology, Division of Toxicology, Central Drug Research Institute (CSIR), Lucknow, India; University of California San Francisco, United States of America

## Abstract

**Background:**

*Caenorhabditis elegans* has emerged as a very powerful model for studying the host pathogen interactions. Despite the absence of a naturally occurring viral infection for *C. elegans*, the model is now being exploited experimentally to study the basic aspects of virus-host interplay. The data generated from recent studies suggests that the virus that infects mammalian cells does infect, replicate and accumulate in *C. elegans*.

**Methodology/Principal Findings:**

We took advantage of the easy-to-achieve protein introduction in *C. elegans* and employing the methodology, we administered HIV-1 protein Nef into live worms. Nef is known to be an important protein for exacerbating HIV-1 pathogenesis in host by enhancing viral replication. The deletion of *nef* from the viral genome has been reported to inhibit its replication in the host, thereby leading to delayed pathogenesis. Our studies, employing Nef introduction into *C. elegans*, led to creation of an *in-vivo* model that allowed us to study, whether or not, the protein induces effect in the whole organism. We observed a marked lipodystrophy, effect on neuromuscular function, impaired fertility and reduced longevity in the worms exposed to Nef. The observed effects resemble to those observed in Nef transgenic mice and most interestingly the effects also relate to some of the pathogenic aspects exhibited by human AIDS patients.

**Conclusions/Significance:**

Our studies underline the importance of this *in vivo* model for studying the interactions of Nef with host proteins, which could further be used for identifying possible inhibitors of such interactions.

## Introduction

Human Immunodeficiency Virus -1 (HIV-1) Nef is accessory protein, found early after HIV-1 infection in the cell, even before the virus is integrated [1] and it regulates multiple functions in host that enhance HIV-1 pathogenesis. The known functions that Nef regulates are down regulation of the surface receptors CD4 [2] and MHC-I [3], ability to modulate T cell signalling [4], [5] and anti-apoptosis [6].

All the stated functions are genetically separable from each other and are regulated by Nef-host protein interaction. Nef mediated MHC-I down regulation is initiated by interacting with host PACS-2. The Nef/PACS-2 complex localize to trans-Golgi network (TGN) where it binds to src family kinase (SFK). The SFK would then bind and phosphorylate ZAP70/Syk on tyrosine enabling ZAP70/Syk to bind the SH2 domain of phosphatidylinositide 3-kinase (PI3K). The activated PI3K then leads to elevated PIP3, stimulation of the guanine nucleotide exchange factor ARNO, and GTP loading of ARF6. During the pathway, the ternary complex of Nef engage with other proteins PACS-I, AP1 and MHC-I for transit of MHC-I from the plasma membrane to an internal compartment in a paranuclear region [7]–[11]. The Nef induced CD4 downmodulation is mediated by a key molecular heterotetrameric clathrin-associated adaptor protein 2 (AP2). For CD4 down regulation, the direct binding of NEF with AP2 is with dileucine motif 160EXXXLL165 and two acidic residues 174E/DD175 in Nef, which is found in a flexible loop ranges from 148 to 180 amino acids. Mutations of either the dileucines or the diacidic residues to alanines disables Nef binding to AP2 in yeast three hybrid assay and inhibits CD4 down regulation function [12], [13]. Moreover, Colmen et al [14], proposed an alternative pathway which is AP-2 independent pathway and showed direct binding of Nef to the cytoplasmic tail of CD4. The role of Nef in activating T cells is shown by interacting with signalling proteins acting in the T cell receptor(TCR) environment such as LCK, Vav, PAK and PKCθ [4]. Their interaction could be possible in glycolipid–enriched microdomain (lipid rafts) where Nef is found [15]. Nef forms complex with Pak2 [16]–[19] and activates Pak 2 kinases [20], [21]. An interaction domain that is responsible for Pak2 activation has been observed to include residues 89 and 191 [22]. In myloid lineage Nef activates myloid lineage specific tyrosine kinase HCK. Nef tightly binds to the HCK SH3 domain in vitro and activates its kinases activity [23]. The Nef/HCK interaction is mediated by an SH3 binding domain in Nef 72PQVPLR77 which may be too similar with cellular SH3 interaction. The role of Nef in anti-apoptotic function is due to functional interaction with ASK-1 which links both the Fas- and the TNFR- mediated signals (by Fas ligand and TNFα) to the downstream JNK/p38 pathway [6], [24].

The overall Nef functions were vindicated in HIV-1 pathogenesis when Nef deleted HIV-1 infected patients showed delayed progression towards Acquired Immuno Deficiency Syndrome (AIDS) which had undetectable/low viral load with normal CD4 count [25], [26]. In the experimental model, Nef deleted SIV_mac 239_ upon infection in rhesus monkey showed low viral load, normal CD4 count and monkey lived healthy for long time [27]. These studies suggested that Nef plays role in enhancing viral replication, whether it has direct or indirect effect is not clearly understood. To study the individual function of Nef, pertaining to HIV-1 replication, no feasible, laboratory model is present.

Interestingly, in mice model it was shown that among HIV proteins, Nef harbours a major disease determinant and Nef expressing in CD4+Tcells and macrophage/dendrite lineages develop severe AIDS-like pathogenesis: failure to thrive/weight loss, diarrhoea, wasting, premature death, thymus atrophy, loss of CD4+ T cell, interstitial pneumonitis, and tubulo-interstitial nephritis. Related to molecular function, Nef exhibit constitute tyrosine phosphorylation of LAT and p42/44 MAP kinase and anti-CD3 hyperactivity [28]. All the AIDS-like pathogenic effect in mice is accrued possibly by the interaction of Nef-host protein followed by activating pathways [29]. The Nef-host protein interactions that are initiating pathways cannot be studied easily in mice model because of the complexity of model and limitations in scientific technologies, for example, genetic approaches of the interaction in whole animal cannot be studied by RNA interference (RNAi) techniques.

In recent years, model system *Caenorhabditis elegans (C. elegans)* has been successfully employed for understanding numerous key biological processes. Its appreciable homology of gene sequences with those of humans, its conservation of many disease pathways with higher vertebrates combined with its well studied developmental biology and experimentally manipulable genetics, makes *C. elegans* a precious model system for understanding intriguing questions that relate to human health and disease [30]. In the past, research studies exploiting the strengths of *C. elegans* have achieved significant understanding of the disease conditions like Diabetes, Obesity, Aging, Cancer, Neurodegenerative diseases, Ionchannelopathies and Innate immunity related disorders [31]. Whereas the transgenic, knockout or over-expressing disease models of *C. elegans* have led researchers to identification of important pathways and/or specific genetic targets, the basic research studies aimed at understanding the biological processes have created a wealth of knowledge that directly or indirectly leads to understanding human disease conditions. *C. elegans* has also proved to be a very powerful model for studying of host pathogen interactions. Despite the absence of a naturally occurring viral infection for *C. elegans*, the model is now being exploited experimentally to study the basic aspects of virus-host interplay [32]; [33]. The data generated from recent studies suggests that the virus that infects mammalian cells does infect, replicate and accumulate in *C. elegans*. Using transduction techniques of individual proteins, HIV protein Tat has been shown to be successfully transduced within *C. elegans* [34]

In the present study we transduced viral Nef protein in *C. elegans* and studied the presence and effect of Nef protein in whole animal. Our result showed that transduced Nef protein was present in entire tissues of animals and induced several altered physiological effect in animals. These effects observed in *C. elegans* were lipodystrophy, impaired locomotion, compromised reproductive performance and reduced life span of nematodes. These physiological effects in *C. elegans* are comparable with some pathologic symptoms induced in Nef-transgenic (tg)-mice and in HIV-1 patients.

## Materials and Methods

### *C. elegans* culture and maintenance

The studies were carried out in the wild type strain of *C. elegans* (N2, var. Bristol), reared at 22°C on Nutrient Growth medium (NGM) agar plates as described by Brenner [35]. The plates were seeded either with “C41 strain of *E.coli*” (Control) or with “C41 strain of *E.coli* transfected with Nef” (Nef treated) as described in next subsection. For analyses carried out on Day 8 of nematode age, NGM was mixed with FUDR for restricting the growth of progeny on the plates.

### Treatment of nematodes with HIV-1 protein Nef

An inducible sequence of HIV-1 protein Nef was transfected into the C41 strain of *E. coli*. The expression of Nef was induced by adding Isopropyl β-D Galactopyranoside (IPTG) to the liquid culture media at a concentration of 100 mM. The bacterial cultures were plated onto the NGM plates, incubated overnight (approx 18 hours) at 37°C. On the day of initiation of treatment, gravid nematode populations were subjected to axenization for isolation of embryos [36] so as to have a synchronous population of nematodes. The isolated embryos were then transferred to plates seeded either with *E. coli* strain C41 (control group) or with *E. coli* strain C41 transfected with HIV-1 Nef sequence (Nef treated group).

### Immunohistochemistry using anti-Nef antibody

In order to confirm the absorption of Nef inside the system, Immunohistochemistry of Nef treated nematodes was carried out using an anti-Nef primary antibody and Alexa Fluor 488 secondary antibody. The method described in [37] was followed with minor modifications. Briefly, the synchronous populations of nematodes raised either on C41 or on Nef transfected C41, were washed off the plates using M9 buffer. The adhering bacteria were cleaned off by washing thrice with M9 buffer with brief centrifugations at 100 g in between each washing. The washed worms were fixed with 2.5% glutaraldehyde for 1 hour, freeze fractured and permeabilized using Phosphate buffered saline with Triton X-100 (PBST) overnight. The premeabilized worms were then incubated in 1% BSA for one hour followed by subsequent incubations in anti-Nef primary antibody at 1∶100 dilution (3 hours) and Alexa Fluor 488 (Invitrogen) secondary antibody at 1∶1000 dilution (for 1 hour). The worms were finally mounted on slides using Prolong antifade mounting medium (Invitrogen), sealed using DPX and analyzed for staining pattern under laser scanning confocal microscope (Carl Zeiss).

### Phenotypic analysis for studies on lipodystrophy

The phenotypic analysis of lipodystrophy induced by Nef was carried out using stereozoom lab microscope. The thickness of Nef treated worms was compared to that of control subjects. The overall thickness of nematodes was quantified from still images, in terms of total worm area using Image J software (Image J, National Institutes of Health, Bethesda, MD).

### Nile Red staining of lipid deposits in nematodes

The method described by Ashrafi *et al* [38] was followed. Briefly the non-toxic lipid specific dye- Nile Red (Sigma), was mixed with *E.coli* before seeding it onto the NGM plates/NGM-FUDR plates. The synchronous aged embryos, derived from axenizing by hypochlorite treatment, were transferred onto the seeded plates. Nematodes were allowed to grow on normal NGM plates for 48 hours and on NGM-FUDR plates for 8 days. At the termination of treatment, nematodes were washed off the plates using M9 buffer, cleared off any adhering bacteria by 2–3 washings and mounted in sodium azide/glycerol mixture, using agar padded cover slip on a glass slide. The slides were sealed using DPX mountant and analyzed using laser scanning confocal microscope (Carl Zeiss). The still images were processed using Image J software (Image J, National Institutes of Health, Bethesda, MD) for quantification of florescence intensity.

### Thrashing assay for effect on motility of nematodes

The thrashing assay of *C. elegans* is a well described method to measure their motility. In this assay, control and treated worms were washed off their parent plates using M9 buffer. The worms were washed 2–3 times for getting rid of any adhering bacteria. A drop of M9 buffer was placed on a clean glass slide. A single worm was placed in the drop of buffer and allowed to stabilize for 30 seconds. The slide was placed under a stereozoom microscope, the timer was set at 30 seconds and the frequency of sigmoidal body bends was counted. One thrash was defined as complete bending of the worm body one way to the outermost angle and back to the initial posture. Thrashes were counted from 10 worms per treatment condition.

### Aldicarb assay for effect on excitatory neurotransmission

Aldicarb purchased from Supelco was stored as 1 M stock in 70% ethanol, at 4°C. NGM plates for the aldicarb assay were prepared by adding aldicarb stock to the medium, prior to pouring onto plates, so as to achieve a final concentration of 1 mM. The assay plates were stored at 4°C and used within two weeks of preparation. For carrying out the assays, the aldicarb assay plates were bought to room temperature. The control or treated worms were washed off their parent plates, cleared off the adhering bacteria and transferred onto the aldicarb assay plates (30–40 worms per plate). The paralysis phenotype of worms was scored at a fixed time point of 3 hours and compared to that of control worms.

### Assay for quantification of effect on reproductive performance of nematodes

The effect of Nef on reproductive performance of *C. elegans* was analyzed by rearing the synchronous populations of worms on control or Nef transduced *E. coli*. At 48 hours of treatment, 10–20 worms were transferred from parent plates to fresh plates of the same treatment condition. Worms were reared on the fresh plates for another 24 hours. Experiments were carried out in triplicates for each treatment condition and the total number of worms laid on each plate per 24 hours was recorded to calculate the total number of eggs laid per worm per 24 hours (fecundity), as a measure of the reproductive performance of the nematodes. The parent worms were removed from the plates and the progeny arising from the eggs laid was analyzed the next day to quantify the hatchability of laid eggs.

### Life span assay

The effect of Nef on life span of nematodes was analyzed by raising the worms on normal or Nef treated medium right from the embryonic stage (embryos isolated as per [10]). The worms were transferred to fresh plates everyday for first ten days and every alternate day thereafter. This was done in order to prevent mixing of two generations. The number of dead worms was counted each day until the last worm was dead.

### Statistical analysis

Statistical analysis was carried out using Sigma Stat software package; calculation of statistical significance between various groups was carried out employing Student's t test.

## Results

### Trans-cellular introduction of HIV-1 protein Nef in *C. elegans*

**Figure 1:**
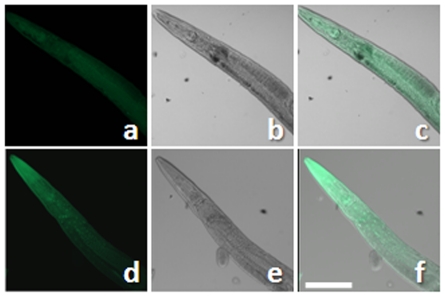
Representative images for Immunohistochemical staining of freeze-fractured *C. elegans* stained for the presence of viral protein Nef on third day of treatment. a, b and c - Control, d, e and f - Nef treated. Images a and d are fluorescent images, b and e are images grabbed using Differential Interference Contrast (DIC) optics, c and f are merged images of a,b and d,e respectively. Scale bar, 50 µm.

The feeding of worms on bacteria, transformed with an inducible sequence of Nef, resulted in absorption of Nef via the gut of nematodes. The Immunohistochemistry of nematodes exposed to Nef for a period of 48 hours showed significant presence of Nef in the worm. More than 90% of the Nef treated nematodes exhibited a positive staining pattern with a variability of less than 10%. Worms were imaged after probing with an anti-Nef primary antibody and Alexa Fluor 488 (Molecular Probes) secondary antibody Fig. 1 (a and d) show representative fluorescence images of control and Nef treated nematodes respectively. Figure 1b is Differential Interference Contrast (DIC) image and Fig. 1c is merged image of Fig. 1a and b. Similarly Fig. 1e and Fig. 1f are DIC and merged images for Fig. 1d. Such transcellular introduction has been shown for another HIV-1 protein, Tat, in *C. elegans* [34]. Previously, feeding of *C. elegans* on bacterial strains expressing dsRNA sequences, has been successfully employed to achieve systemic absorption of the dsRNA, leading to RNAi mediated silencing of the target gene in the nematode [38].

### Nef induces severe lipodystrophy in *C. elegans*

**Figure 2:**
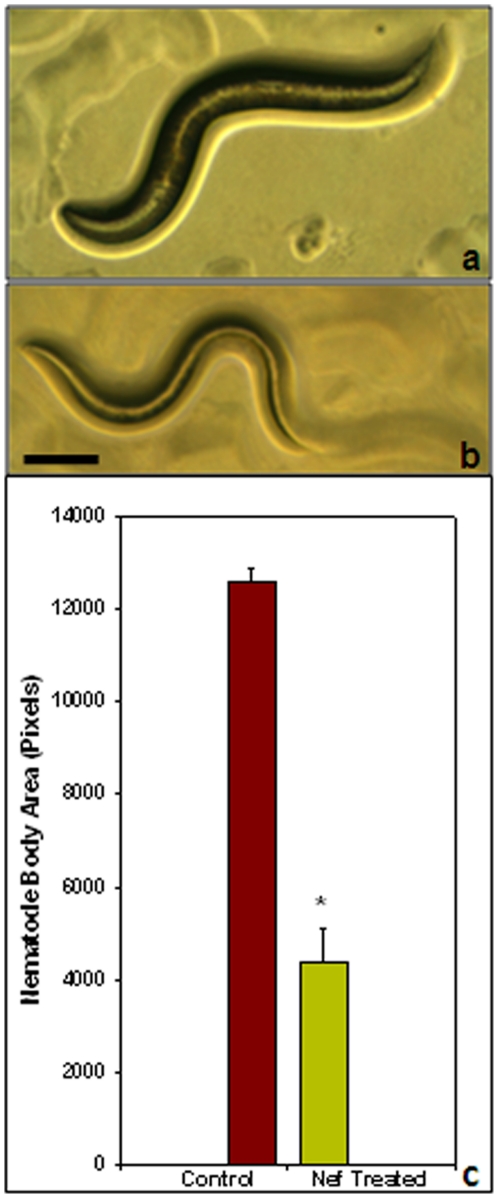
A representative phase contrast image of live worms showing normal (a) and thin Nef treated nematodes (b) on day 8 of treatment. Scale bar, 100 µm. Fig. 2c is the graphical representation of the ‘total body area’ of nematodes (*p<0.05).

**Figure 3:**
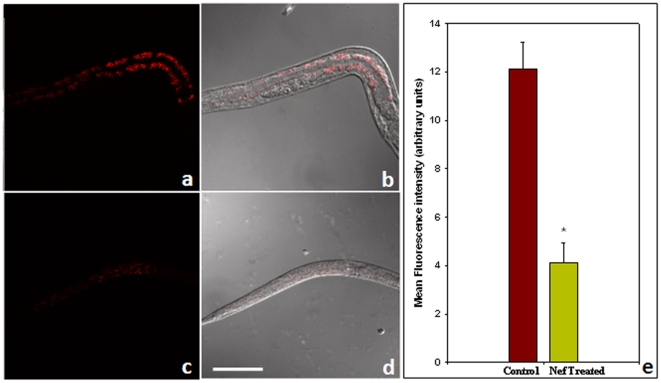
Representative images for Nile red staining of Control (a,b) and Nef treated (c,d) worms (on day 8 of treatment) showing the reduced amount of lipid deposits in Nef treated worms as compared to Control. (a) and (c) are fluorescence images, (b) and (d) are merged images from DIC and fluorescent microscopy. Scale bar, 50 µm. Fig. 3e is graphical representation for fluorescence intensity of the nematodes as quantified using Image J software (*p<0.05).

One of the very striking observations made after the onset of adulthood of nematodes, was that of severe lipodystrophy. More than 85% of the worms raised on Nef loaded bacteria developed into thin adults (Fig. 2b) as compared to their control counterparts (Fig. 2a). The thickness of the worms, quantified in terms of ‘total body area’ was found to be 12604.5±254.5 pixels in control worms, whereas Nef treated worms were found to have a total body area of 4391.0±739.0 pixels; thus exhibiting statistically significant (p<0.05) reduction (Fig. 2c). The reduced thickness in Nef treated worms was further confirmed to be as a result of reduced lipid deposition, by staining the worms with lipid specific dye nile red. Fig. 3a and 3c show representative images of the staining pattern of Control and Nef treated worms respectively, Figures 3b and 3d being the merged pictures for fluorescence and DIC images of control and Nef treated groups respectively. The staining intensity of Nef treated worms, as quantified by Image J software, was found to be 4.11±0.82 arbitrary units, which was significantly (p<0.05) lower than that of control worms which exhibited a fluorescence intensity of 12.12±1.09 arbitrary units (Fig. 3e). This observation of lipodystrophy is very much in agreement with the effect of HIV pathogenesis in humans, as the virus is known to induce multiple abnormalities including insulin resistance, lipodystrophy and weight loss. This is because HIV replication induces cellular enzymes and proteins that are significantly associated with biologically relevant processes involved in lipid synthesis, transport and metabolism.

### Nef effects motility of worms by reducing excitatory neurotransmission

**Figure 4:**
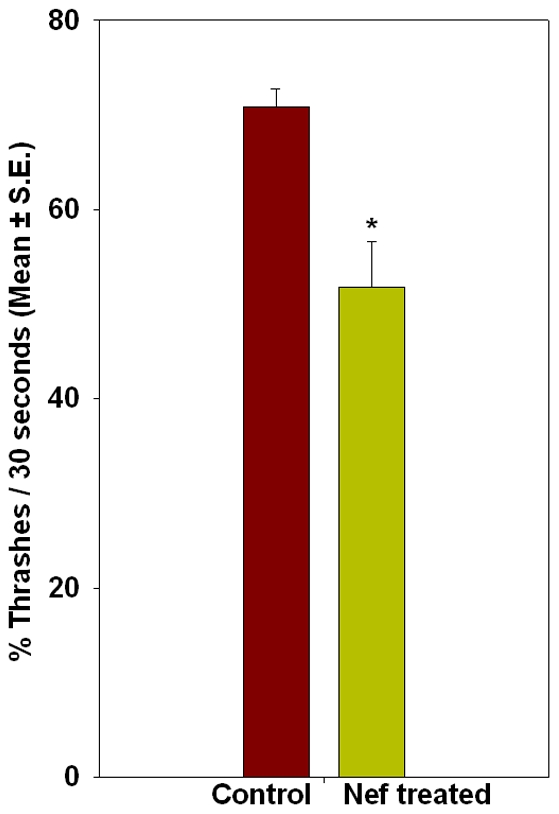
Histogram showing effect of Nef on thrashing in C. elegans; *p<0.005.

**Figure 5:**
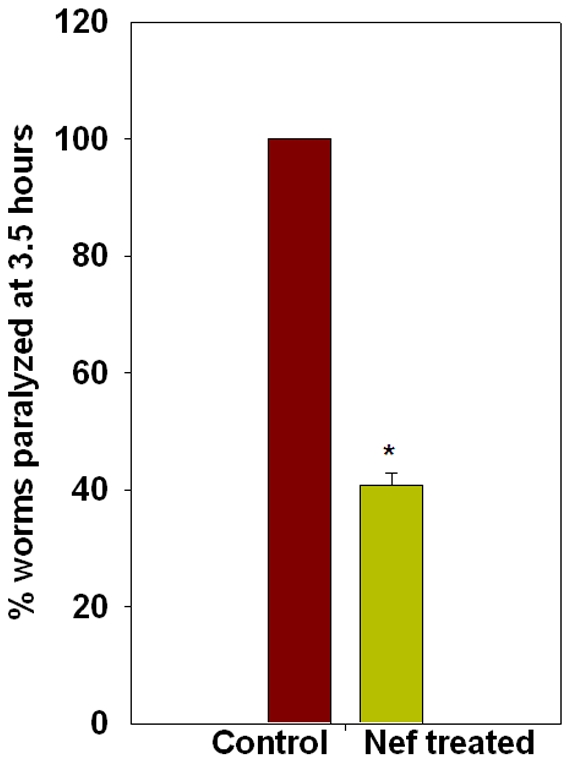
Histogram showing effect of Nef on Aldicarb induced paralysis; *p<0.005.

Nematodes exposed to Nef for longer durations exhibited an impaired gait and reduced locomotion. On an agar surface plated with bacteria *E. coli*, worms were unable to express their typical sinusoidal waves. We carried out thrashing assays of worms on day 3 and day 8 of the treatment. This was based on our phenotypic analysis wherein we figured that effect of Nef is more pronounced after at least 8 days of treatment. Thrashing in a drop of buffer, was reduced in worms treated with Nef for 8 days as compared to the control worms of same age (Fig. 4). Control worms exhibited a thrashing count of 71 thrashes per 30 seconds, whereas the thrashing count of Nef treated worms was 52 thrashes for the same duration. Thus Nef treatment was observed to induce a statistically significant (P<0.005) 27% reduction of thrashing in nematodes. The behavioral pattern of lateral swimming or thrashing is exhibited by nematodes while placed in any liquid. A healthy worm thrashes continuously until exhausted of energy or until moved back to a solid surface. However, if the neuromuscular function of the nematode is compromised by exposure to any form of stress, the effect reflects on the thrashing count too. In the present experiment the Nef treatment seems to have affected the neuromuscular function that resulted in the observed effect. To further confirm the effect of Nef on neuromuscular function, we carried out an assay employing Acetylcholine esterase (AChE) inhibitor aldicarb. The exposure of healthy nematodes to aldicarb results in reduced availability of AChE at the neuromuscular junctions of worms, thereby leading to an increased accumulation of neurotransmitter acetylcholine (ACh) at the neuromuscular junction (NMJ), which in turn leads to paralysis of nematodes. Depending upon increasing or decreasing the availability of ACh at the NMJ, any chemical treatment or mutation, hastens or delays the induction of this paralytic effect. In the present study, exposure of nematodes to Nef for longer duration resulted in delaying of aldicarb induced paralysis. The worms assayed for aldicarb induced paralysis on Day 3 did not show a statistically significant effect, whereas the worms assayed on day 8 of treatment showed a 59% resistance with respect to control worms (Fig. 5). In both the conditions worms were raised on Nef right from the embryonic stage.

### The reproductive performance of *C. elegans* is impaired by Nef

**Figure 6:**
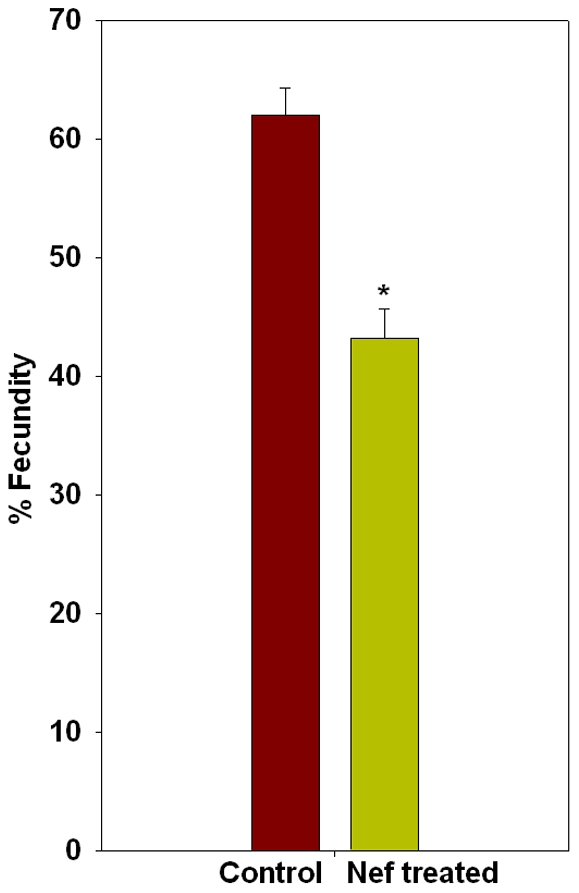
Histogram showing effect of Nef on fecundity of *C. elegans*; *p<0.005.

**Figure 7:**
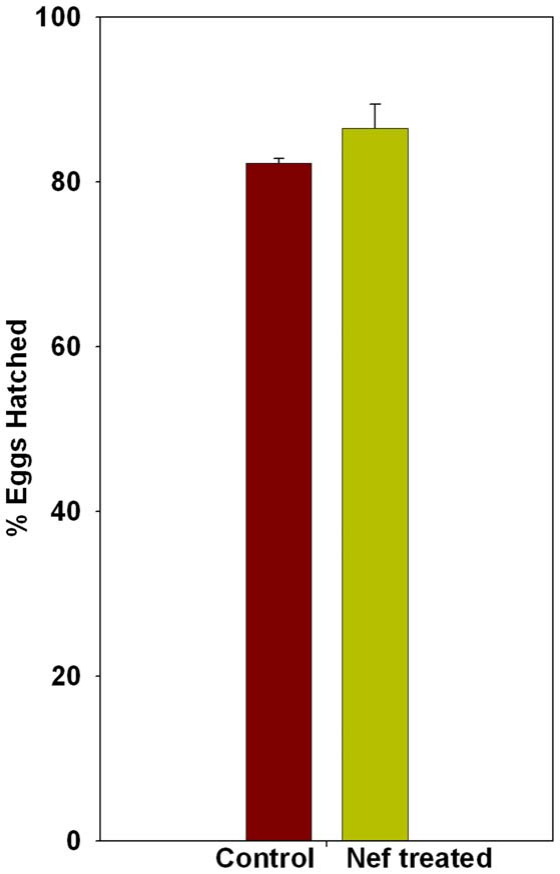
Histogram showing effect of Nef on hatchability of eggs in *C. elegans*.

Each hermaphrodite nematode is known to lay approximately 300 eggs during its entire life span. In particular, egg laying starts at the onset of adulthood and most number of eggs are laid during the early half of the adult life of hermaphrodites. In the present study, we observed a significant reduction of egg laying in Nef treated worms when compared to their control counterparts observed for their fecundity for a period of 24 hours starting at 48 hours of their age. The average number of eggs laid by worms of control group was 61.9±2.2, whereas the average for Nef treated worms was 43.2±2.4, thus Nef treated worms exhibiting a 30.2% reduction (P<0.005) in number of eggs laid in 24 hours (Fig. 6). The laid embryos, however, did hatch normally and the average percentage of hatched eggs was not statistically different between control (82.2±0.6) and Nef treated (86.4±2.9) worms (Fig. 7).

### Nef induces early mortality in exposed *C. elegans* populations

**Figure 8:**
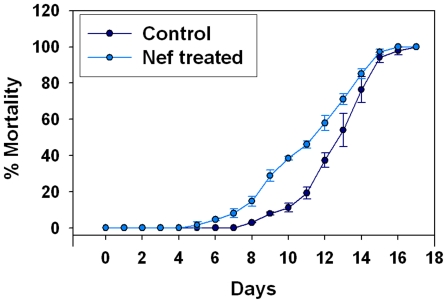
Line graph showing effect of Nef on the life span of *C. elegans*.

To study the effect of Nef on longevity, we raised the nematodes on Nef loaded bacteria and observed effects on their life span as compared to the control worms. Our studies showed that Nef induced early mortality in the exposed population. On day 10 the percentage mortality in control group was 11.2±2.5, whereas Nef treated group exhibited a mortality percentage of 38.5±0.9 (Fig. 8). The last mortality in control group occurred on day 17, whereas all the worms of Nef treated group had died by 16^th^ day.

Significantly, another HIV protein Tat, was not shown to induce any pathogenic effects in *C. elegans* [34]. Further our studies with another protein, PACS1, transfected into *C. elegans* did not induce any pathogenic response in the nematodes. This underlines the fact that observed effects are caused specifically by Nef in the treated nematodes.

## Discussion

Our results show that Nef protein was successfully transduced into the *C. elegans* system. The effects that Nef induced in the nematode include; lipodystrophy, altered locomotion, compromised reproductive performance and reduction of life span. These effects, observed in *C.elegans* are well comparable with some pathologic symptoms induced by Nef in transgenic mice and in HIV-1 patients. Since, Nef does not have any enzymatic activity, the functions scored by Nef are only by Nef-host protein interaction [39]. Likewise, Nef-tg-mice showed AIDS like pathogenesis and inhibition of CD4 downregulation which has been attributed to the Nef-host protein interaction [40].

The worms treated with Nef protein developed into thin adults. Staining of the Nef treated nematodes with lipid specific stain, Nile Red, showed that lipid deposition in the Nef exposed population was significantly reduced. Previously, body weight loss has been reported in mice expressing Nef protein [40]. Presently, lipodystrophy in HIV-1 patient is extensively studied with the usage of different anti-retro viral drugs (HAART) and the molecular mechanisms affected in lipid metabolism during HAART regime is studied [41]. However, the viral proteins involved in fat metabolism were studied in mice [42] but their effect in human patients is not studied pertaining to limitations. In recent years significant efforts have been made to understand the basic mechanism of fat accumulation in mammalian cells. The molecular pathways and genes involved in regulating fat metabolism are conserved between *C.elegans* and mammals [43] For example, there is evidence to support the existence of neuropeptide, serotonergic, and insulin signaling pathways that regulate fat storage in both nematodes and mammals [44–46]. Specifically, deciphering the pathways and genes affected by Nef in *C.elegans* may be helpful to understand the molecular pathways regulating HIV lipodystrophy which is characterized by hypertriglyceridemia, low HDL cholesterol, high total cholesterol, insulin resistance, altered energy expenditure and lipomatosis [47].

In our studies Nef was observed to effect normal locomotory behavior as evidenced by reduced thrashing of treated worms. The overall phenotypic analysis and the severe effect on thrashing pointed towards effect of Nef on excitatory neurotransmission. To understand this, we carried out a pharmacological assay employing an Acetylcholine Esterase (AChE) inhibitor aldicarb. Our observation of increased resistance to paralysis in case of worms treated with Nef made us believe that the viral protein affects the availability of excitatory neurotransmitter Acetyl Choline (ACh) at the synapse. This could be either because of reduced synthesis of the neurotransmitter ACh or because of its increased breakdown before its reaching the synapse. The indication of such an effect of Nef on the availability of ACh may give us lead to understand and identify the molecular mechanism of Nef induced effect on ACh-AChE pathway, as previous reports have also shown AChE mediated CD4+T cell activation in response to HIV infection [48].

In our studies the effect of Nef was also observed on the reproduction of *C.elegans*; an effect not reported previously in other model systems probably because other effects of the viral protein, including effect on longevity, are so pronounced that the effect on reproduction cannot be studied. Model system *C. elegans* made it easy to study the effect as it attains sexual maturity early on, even before other effects are well pronounced. Our studies show that Nef significantly reduces fecundity of the exposed nematodes; though the laid embryos do hatch normally, but the overall brood size of the exposed population was significantly reduced as a result of Nef exposure. Previously various chemical effects have been reported to affect *C. elegans* reproduction as a result of their toxic effects [49, 50]. In the current study, Nef might also be inducing deleterious effects on the pathways that control the nematode reproduction. Interestingly a recent report has also reported role of a common factor, Klf-3, in altering lipid deposition and affecting reproduction of *C. elegans* [51]. It could very well be possible that a similar effect is being induced by Nef that is altering the downstream signaling pathways leading to the observed phenotypes.

*C. elegans* is a well studied model for ageing; studies on protein interaction networks have shown conservation of ageing process between humans and other invertebrate species including C. *elegans* [52]. The overall pathological effect of Nef observed in the present study has cumulative effect on decreasing the life span of nematodes. Most notably, this effect in the present study was observed in the absence of any confounding factors like opportunistic infections that, otherwise, are known to induce early mortality in case of HIV patients. In the present study, the effect of Nef on life span indicates that this critical viral protein *per se* does induce adverse effects as drastic as reducing longevity of the exposed organism. Similarly in mice, Nef transgenic mice lines, have reduced life span compared to control [40]. These observations, from model system *C. elegans*, make it tempting to speculate that the presence of Nef not only enhances the viral replication thereby increasing the viral load in host, but also contributes towards an increased pathogenesis leading to multiple pathological effects. This model will be immensely useful towards identifying specific host-pathogen, protein-protein interactions and towards further deciphering of molecular mechanisms of Nef pathogenesis in HIV-1 infected patients. The model would further be useful towards screening of specific inhibitors as also suggested previously via virtual screening of first set of possible inhibitors [53].

Our studies lead towards creation of an in vivo environment, in the genetically relevant and experimentally accessible model system *C. elegans* which would be of immense importance for studying the interactions of Nef with host proteins and would further be used for identifying possible inhibitors of such interactions.
